# Entropy Analysis of Temperature Swing Adsorption for CO_2_ Capture Using the Computational Fluid Dynamics (CFD) Method

**DOI:** 10.3390/e21030285

**Published:** 2019-03-15

**Authors:** Zhihao Guo, Shuai Deng, Shuangjun Li, Yahui Lian, Li Zhao, Xiangzhou Yuan

**Affiliations:** 1 Key Laboratory of Efficient Utilization of Low and Medium Grade Energy (Tianjin University), Ministry of Education of China, Tianjin 300350, China; 2 International Cooperation Research Centre of Carbon Capture in Ultra-Low Energy-Consumption, Tianjin 300350, China; 3 Department of Chemical and Biological Engineering, Korea University, 145 Anam-ro, Seongbuk-gu, Seoul 02841, Korea

**Keywords:** carbon capture, temperature swing adsorption, CFD, entropy generation, heat regeneration, non-equilibrium thermodynamic

## Abstract

Carbon capture by adsorption is supposed to be an effective method to reduce CO_2_ emissions, among which Temperature Swing Adsorption (TSA) can utilize low-grade thermal energy even from renewable energy source. At present, TSA technology still has several challenges to be practical application, such as intensive energy-consumption and low energy-efficiency. Thermodynamics could be a powerful method to explore the energy conversion mechanism of TSA, among which entropy analysis could further provide a clear picture on the irreversible loss, even with a possible strategy of energy-efficient improvement. Based on the theory of non-equilibrium thermodynamics, the entropy analysis of TSA cycle is conducted, using the Computational Fluid Dynamics (CFD) method. The physical model and conservation equations are established and calculation methods for entropy generation are presented as well. The entropy generation of each process in cycle is analyzed, and the influence from the main parameters of desorption process is presented with optimization analysis. Finally, the performance of the cycle with regeneration is compared with that of the cycle without regeneration, and the method of reducing the entropy generation is obtained as well. This paper provides possible directions of performance improvement of TSA cycle with regards on energy utilization efficiency and the reduction of irreversible loss.

## 1. Introduction

The Intergovernmental Panel on Climate Change (IPCC) claims that human activities are estimated to have caused approximately 1.0 °C of global warming above pre-industrial levels and will lead to serious ecological consequences if this increase continues [1]. Global warming is mainly caused by greenhouse gases, among which CO_2_ is the primary contributor, and thus CO_2_ emission reduction is extremely urgent [2]. Carbon capture technology, which is an effective way to reduce carbon dioxide emission, commonly includes pre-combustion carbon capture, oxy-combustion carbon capture and post-combustion carbon capture [3]. Post-Combustion Carbon Capture has the advantages of easy integration into existing power plants and convenient maintenance [4]. Furthermore, the post-combustion trapping techniques mainly include absorption, adsorption, membrane separation, cryogenic separation and so on [3,5]. Carbon capture by adsorption has been widely concerned due to its advantages of being applicable to a wide range of temperatures and being used for air capture [6]. Carbon capture by adsorption includes temperature swing adsorption (TSA), pressure swing adsorption (PSA) and electricity swing adsorption (ESA), etc. Among those, TSA technology has a good development prospect because it is easy to integrate with various renewable energy sources for heat-driven and thus make full use of low-grade thermal energy [7,8,9,10].

However, the energy consumption of carbon capture by adsorption is still a major problem that limits its development. There have been several articles concerning the energy consumption analysis of the adsorption separation cycle [6,11,12,13,14]. For VPSA, energy consumption of adsorption separation mainly comes from compressor and vacuum pump, while for TSA and ESA, energy consumption of heating process mainly consists of energy consumption of adsorbent sensible heat and regenerated latent heat.

Recently, Zhao et al. [15] proposed a carbon pump model to evaluate the energy-efficient characteristics of the existing CO_2_ adsorption technologies. Derived from the concept of the second law of thermodynamics, the carbon pump model theory includes minimum CO_2_ separation work and second law efficiency, establishing a relationship between energy consumption and separation difficulty. For an ideal gas mixture and a reversible process with no chemical reaction at constant pressure and temperature, the minimum separation work of the system can be calculated. The efficiency of the second law can be defined based on the ratio of the minimum separation work to the actual input work. Based on the above theory, Zhao et al. carried out theoretical analysis and sensitivity analysis of process parameters for TSA [16], VPSA [17,18], PTSA [17] and ESA [19] systems respectively, and obtained a series of qualitative conclusions. The literature-research results show that the second law efficiency of carbon capture by adsorption is mainly concentrated in the range of 10–20%. The current second law of adsorption carbon capture system is generally low (<20.77%), and there is still a large potential for improvement [15].

**Figure 1:**
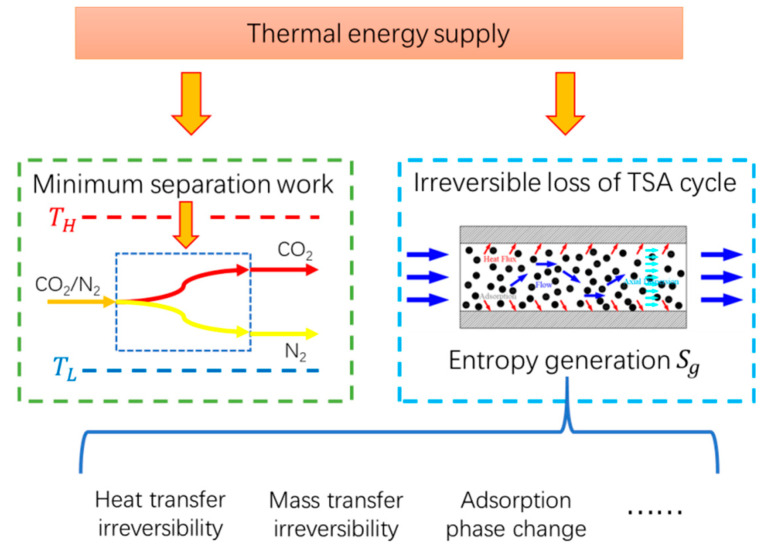
The role of entropy analysis for TSA.

According to theory of classical thermodynamics, the difference between actual energy consumption and theoretical energy consumption is due to the irreversibility in the entire adsorption system, which can be expressed by the entropy generation in the adsorption cycle [20]. Therefore, it is very important to conduct the entropy analysis of adsorption separation cycle for TSA, as shown in Figure 1, as an effective make-up to the existing researches mainly on energy-balance analysis. At present, there are few papers concerning entropy analysis of adsorption separation process, especially on carbon capture by adsorption. Myat et al. [21] conducted entropy analysis on the adsorption dehumidification process driven by low-temperature waste heat, and calculated the entropy generation of the adsorption process and the heating desorption process. The calculated entropy generation includes entropy generation of adsorption, entropy generation of heat transfer and entropy generation of water flow in the heating and cooling coil. The one-dimensional model was adopted and the heating temperature was optimized. Li et al. [22] analyzed the CO_2_ removal devices of manned spacecraft and assumed that entropy generation was mainly generated by heat transfer, and used entropy generation as one of the objectives for multi-objective optimization. As one of the important applications of adsorption phenomenon, adsorption refrigeration cycle is very similar to adsorption separation cycle. Many papers [23,24,25,26,27] have calculated the entropy generation in the process of adsorption refrigeration, with calculation method similar to Myat et al. [21]. But there is a difference between the adsorption refrigeration cycle and the adsorption separation cycle, mainly because the adsorption refrigeration cycle is single component adsorption and the cycle configuration between them is quite different. In the existing studies, entropy analysis mainly adopts the one-dimensional or lumped calculation method, when two-dimensional or above model, which can be done using the CFD approach, can achieve higher accuracy and can be applied to the optimization analysis of complex adsorption bed. In addition, the higher dimensional model can be used to calculate the irreversibility within the adsorption bed more accurately (such as considering the irreversibility of the radial transfer process). In addition, there is a lack of comprehensive analysis of the entropy generation of various irreversibility factors in the adsorption and separation cycle, so as to fully consider the irreversible loss in the adsorption and separation process. According to the theory of non-equilibrium thermodynamics, entropy generation can be expressed as the product of thermodynamic flow and thermodynamic force in the non-equilibrium process. Compared with the Clausius calculation method of entropy [20,28,29,30,31], this calculation method could provide a clearer explanation on the irreversible factors in thermodynamic processes.

In this paper, the CFD method is applied to comprehensively calculate and analysis the entropy generation in the three-step temperature swing adsorption cycle. Firstly, the CFD model is introduced in the second part of this paper, including mesh division, conservation equations and corresponding boundary conditions. And then, the calculation method of entropy generation and energy consumption is also introduced for a post-processing. Then the simulation results are presented in the third part of this paper, including the result of mesh independence verification, entropy generation analysis and parameter sensitivity analysis. The adsorption separation cycle with regenerative process is the research object in the simulation, and the influence of the regenerative process on the entropy generation and energy consumption is analyzed. Finally, the conclusion is given in Section 4.

## 2. Research Methodology

**Figure 2:**
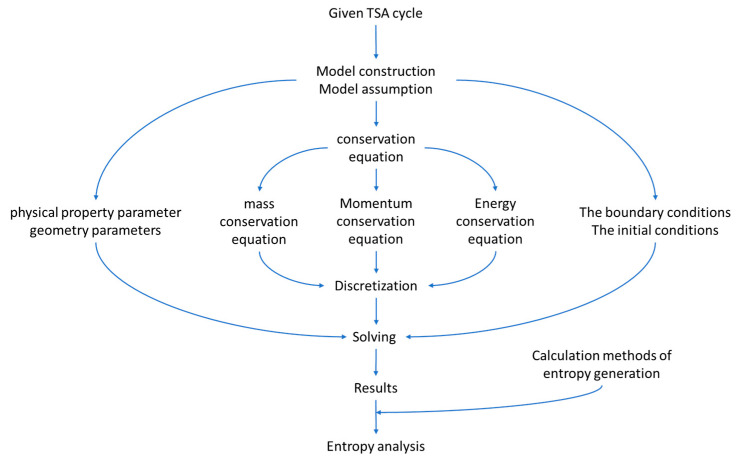
The diagram of simulation process.

In this work, we modeled and simulated the TSA cycle by using the following steps as shown in Figure 2.

### 2.1. Physical Model

In order to study the generation mechanism of irreversibility of TSA cycle, a simple three-step TSA cycle is adopted in this paper, including the following processes:

(1) Adsorption process: the mixture of CO_2_ and N_2_ continuously enters the adsorption bed from the inlet. CO_2_ gas is adsorbed in the adsorption bed through the selective adsorbent material, and N_2_ flows out of the adsorption bed from the outlet.
(2) Desorption process: the adsorption bed is heated, and the adsorbed gas is desorbed and flows out of the adsorption bed.
(3) Cooling process: the adsorption bed is cooled, during which the adsorption bed outlet and inlet are closed.

**Figure 3:**
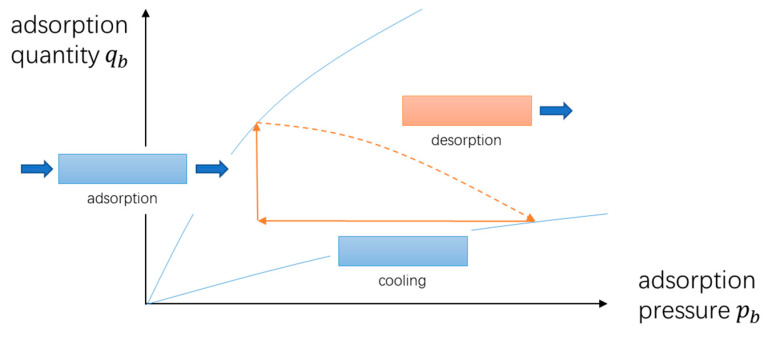
Schematic of the ideal three-step TSA cycle.

The ideal three-step TSA cycle is shown in Figure 3.

The adsorbent material used in this paper is MOF-74, which has the characteristics of large adsorption capacity and strong adsorption selectivity [11,32]. The adsorbent material is a granular porous material filled in the adsorption bed. There are large gaps in the filler layer that allow the mixture to flow through and exchange mass and heat with the adsorbent material.

Adsorption bed is an important part in the cycle of adsorption separation. When the adsorption bed is heated or cooled, the heating method of external fluid convection heat transfer is usually adopted. The outer wall of adsorption bed is usually made of metal materials, and its heat capacity is smaller than that of the external fluid. When the velocity of the external heat transfer fluid is fast, the heat transfer between the adsorption bed and the external fluid has little influence on the fluid temperature. Based on the above discussion, the CFD model adopted in this paper is based on the following assumptions:

(1) The porous media filling layer is regarded as continuous medium.
(2) The mixture of CO_2_ and N_2_ is assumed to be an ideal gas
(3) The flow process is considered as laminar flow
(4) The physical properties of the adsorbent material are assumed to be constant
(5) The mass transfer phenomenon in the adsorption process was described by the LDF model
(6) Coupling effect in heat transfer and diffusion process is not considered
(7) It is assumed that the heat capacity of the metal outer wall of the adsorption bed is very small and negligible, and only the internal process of the adsorption bed is simulated
(8) Assume that the velocity of the external heat transfer fluid is very fast and the heat capacity is very large, and the temperature of the external heat transfer fluid is approximately constant. Therefore, the interior wall temperature of adsorption bed is also assumed to be constant during the process of adsorption, desorption and cooling.

### 2.2. Mesh and Conservation Equation

**Figure 4:**
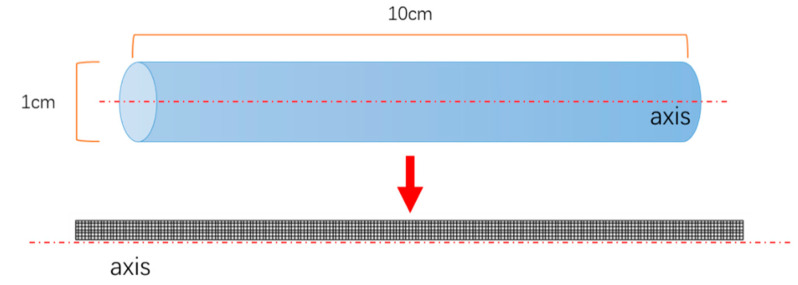
Schematic diagram of 2D grid division.

The adsorption bed used in this paper for irreversibility analysis has a length of 10 cm and an inner diameter of 1 cm. It is divided into grids with a number of 720, 1620, 2880 and 4500, respectively, and the grid independence test is carried out, as shown in Figure 4. The results of grid independence test will be introduced in the third section of this paper.

To simulate the heat transfer, mass transfer and flow process in adsorption bed, complete conservation equations need to be established. The model of Ben-Mansour et al. was adopted for simulation in this paper, of which the accuracy has been verified in their studies [11]. Therefore, the accuracy of the model is no longer verified in this paper. Total mass conservation equation, mass conservation equation and momentum conservation equation can be expressed by Equations (1)–(3):

(1) $$\varepsilon \frac{\partial \rho }{\partial t}+\nabla \cdot (\rho \vec{v})=-(1-\varepsilon )\rho_{p}\sum_{i}^{}M_{i}\frac{\partial q_{i}}{\partial t}$$

(2) $$\varepsilon \frac{\partial (\rho y_{i})}{\partial t}+\nabla \cdot (\rho \vec{v}y_{i})=-\nabla \cdot (\varepsilon \rho D_{i,m}\nabla y_{i})-(1-\varepsilon )\rho_{p}M_{i}\frac{\partial q_{i}}{\partial t}$$

(3) $$\varepsilon \frac{\partial (\rho v)}{\partial t}+\nabla \cdot (\rho \vec{v}v)=-\nabla p+\nabla \cdot \tau +S_{momentum}$$

where *ρ* (kg m^−3^) is the density of gas mixture, *v* (m s^−1^) is the velocity vector, *ρ_p_* (kg m^−3^) is the density of adsorbent particle, *ε* is the porosity of adsorbent layer, *M_i_* (mol kg^−1^) is the molar mass for component *i*, *q_i_* (mol kg^−1^) is the adsorption amount of component *i*, *y_i_* is the mass fraction for component *i* in gas mixture, *D_i,m_* (m^2^ s^−1^) is the effective diffusion coefficient for component *i*, *τ* is the stress tensor, *S_momentum_* is the momentum source term.

The effective diffusion coefficient *D_i,m_* of component *i* can be calculated by Equation (4):

(4) $$D_{i,m}=(1-x_{i})/\sum_{j,j\neq i}^{}\left(x_{j}/D_{i,j}\right)$$

where *D_i,j_* (m^2^ s^−1^) is the diffusion coefficient of component *i* in component *j*, and can be calculated by Equation (5):

(5) $$D_{i,j}=6.7\times 10^{-8}T^{1.83}{\left(\frac{1}{M_{i}}+\frac{1}{M_{j}}\right)}^{1/2}/\left\{p{\left[{\left(\frac{T_{c}}{p_{c}}\right)}_{i}^{1/3}+{\left(\frac{T_{c}}{p_{c}}\right)}_{j}^{1/3}\right]}^{3}\right\}$$

where *T_c_* (K) is the critical temperature, *p_c_* (Pa) is the critical pressure.

The adsorption kinetic model adopts the LDF model, which can be expressed by Equation (6):

(6) $$\frac{\partial q_{i}}{\partial t}=K_{L,i}(q_{i}^{*}-q_{i})$$

where *K_L,i_* (s^−1^) is the adsorption time constant, q_i_^*^ (mol kg^−1^) is the equilibrium adsorption uptake of component *i*.

The source term of momentum equation is calculated by Ergun model, which can be expressed by Equation (7):

(7) $$S_{momentum}=-(\frac{\mu }{\alpha }v+\frac{1}{2}C_{2}\rho |v|v)$$

where *α* (m^2^) is the permeability, and *C*_2_ (m^−1^) is the inertial resistance factor, *μ* (Pa s) is the gas dynamic viscosity.

In order to fully consider the irreversibility in the adsorption bed, the energy conservation equations adopt the non-thermal equilibrium model, and the energy conservation equation of the gas phase and that of the solid phase need to be established respectively, as is shown in Equations (8) and (9) [33,34]:

(8) $$\frac{\partial }{\partial t}(\varepsilon \rho E)+\nabla \cdot [v(\rho E+p)\}=\nabla \cdot (k\nabla T-\sum_{i}h_{i}J_{i}+\tau \cdot v)+(1-\varepsilon )\frac{6h_{f}}{d_{p}}(T_{s}-T)$$

(9) $$\frac{\partial }{\partial t}(\rho_{p}C_{p,s}T_{s})=\nabla \cdot (k_{s}\nabla T_{s})+\rho_{p}\sum_{i}-\Delta H_{i}\frac{\partial q_{i}}{\partial t}-\frac{6h_{f}}{d_{p}}(T_{s}-T)$$

where *E* (J kg^−1^) is the total energy of gas, *k* (W m^−1^ k^−1^) is the thermal conductivity of gas phase, *h_i_* (J kg^−1^) is the enthalpy of component *i*, *J_i_* (kg m^−2^ s^−1^) is the diffusion flux, *h_f_* (W m^−2^ K^−1^) is the heat transfer coefficient between gas phase and solid, *d_p_* (m) is the particle diameter, *T_s_* (K) is the solid temperature, *C_p,s_* (J kg^−1^ K^−1^) is the specific heat capacity of solid, *k_s_* (W m^−1^ K^−1^) is the thermal conductivity of solids and Δ*H_i_* (J mol^−1^) is the heat of adsorption of component *i*. The convection heat transfer coefficient *h_f_* between bulk gas and adsorbent particles can be calculated by Equation (10):

(10) Nu=2.0+1.1Re6Pr1/3=hfdpk

The adsorption equilibrium of CO_2_ and N_2_ adsorption on MOF-74 was described using the Toth model [11], Equation (11):

(11) $$q_{i}^{*}=\frac{q_{m,i}K_{eq,i}y_{i}P}{{\left(1+{\left(K_{eq,i}y_{i}P\right)}^{n_{i}}\right)}^{1/n_{i}}}$$

where *q_m,i_* (mol kg^−1^) is the maximum adsorption uptake of component *i*, *n_i_* is the heterogeneity parameter of component *i* and *K_eq,i_* (Pa^−1^) is the isotherm adsorption constant of component *i* which can be calculated by Equation (12):

(12) $$K_{eq,i}=k_{0}e^{-\frac{\Delta H}{RT}}$$

where *k*_0_ (Pa^−1^) is the adsorption constant at infinite dilution and *R* (J mol^−1^ K^−1^) is the universal gas constant.

The adsorption bed and process parameters used in the simulation in this paper are shown in Table 1, and the adsorbent parameters are shown in Table 2. The conservation equations are solved by commercial software Ansys Fluent and the calculation method of gas physical properties is described in Appendix A.

**Figure 5:**
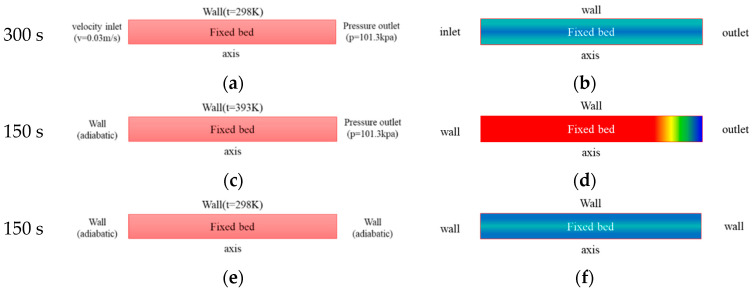
Schematic diagram of boundary condition and initial condition for TSA simulation: (**a**) boundary conditions for adsorption process. (**b**) initial condition for adsorption process. (**c**) boundary conditions for desorption process. (**d**) initial condition for desorption process. (**e**) boundary conditions for cooling process. (**f**) initial condition for cooling process.

The boundary conditions and initial conditions of the three-step TSA cycle simulation are shown in Figure 5. For the boundary conditions, velocity inlet boundary conditions are adopted at the adsorption bed inlet, and pressure outlet boundary conditions are adopted at the outlet. For the initial conditions, the data at the end of the previous process is used as the initial conditions for the next process. The adsorption, desorption and cooling time were 300 s, 150 s and 150 s, respectively.

### 2.3. Computing Method of the Entropy Generation and Energy Consumption

**Figure 6:**
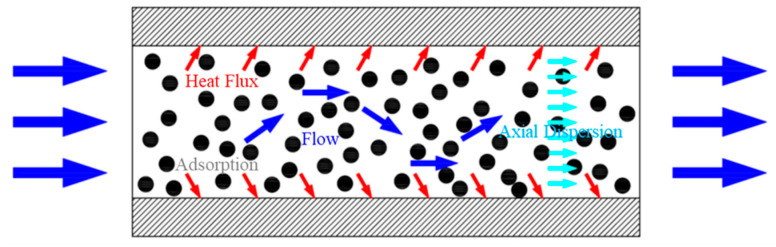
Schematic diagram of adsorption process in adsorption bed.

The transfer process in the adsorption bed includes heat transfer, mass transfer, flow of fluid and adsorption phase transition, as shown in Figure 6. For the non-equilibrium adsorption process, the above process in the adsorption bed has a non-equilibrium potential difference, and the irreversible loss caused by it. The irreversibility of the adsorption process is summarized in Table 3.

In the linear region, the entropy generation can be expressed as a product of thermodynamic forces and fluxes, which can be written as the following equation [35]:

(13) $$\sigma =\sum_{i}J_{i}X_{i}$$

where *σ* (W m^−3^ K^−1^) is the entropy generation rate, *J_i_* is the *i*th thermodynamic fluxes and *X_i_* is the *i*th thermodynamic force. The following is a detailed introduction.

Entropy generation of heat transfer in the fixed bed adsorption include two main part (i.e., entropy generation of heat conduction in the gas phase and the entropy generation of conduction in the solid adsorbent). The entropy generation rate of conduction in the gas phase can be expressed as [35]:

(14) $$\sigma_{q,g}=-\frac{1}{T^{2}}J_{q,g}^{\prime }\cdot \nabla T=-\varepsilon \frac{1}{T^{2}}k{\left(\nabla T\right)}^{2}$$

where *J_q,g_^′^* (W m^−2^) is the heat conduction in the gas phase. Similarly, the entropy generation rate of conduction in the solid phase can be expressed as:

(15) $$\sigma_{q,s}=-\frac{1}{T_{s}^{2}}J_{q,s}^{\prime }\cdot \nabla T_{s}=-(1-\varepsilon )\frac{1}{T_{s}^{2}}k_{s}{\left(\nabla T_{s}\right)}^{2}$$

The entropy generation of gas mixing is caused by diffusion between different components of the gas mixture and can be expressed as [36]:

(16) $$\sigma_{diff}=-\frac{\sum_{i}J_{i,m}\nabla \chi_{i}}{T}=-\varepsilon \frac{\sum_{i}(-\rho D_{i,m}\nabla y_{i})\nabla \chi_{i}}{T}$$

where *J_i,m_* (kg m^−2^ s^−1^) is the diffusion flow of *i* component. And *χ_i_* (J kg^−1^) is the chemical potential of *i* component in gas mixture and can be expressed as:

(17) $$\chi_{i}=(\chi_{i}^{\Theta }+RT\ln x_{i})/M_{i}$$

The entropy generation of frictional flow in the porous media can be expressed as [37,38]:

(18) $$\sigma_{vf}=-\varepsilon \frac{v\cdot \nabla p}{T}$$

The entropy generation between adsorbent and bulk gas due to transport phenomenon is composed of two parts: (1) the entropy generation between adsorbent and bulk gas due to heat exchange caused by temperature difference; (2) the entropy generation of adsorption phase change caused by adsorption or desorption of adsorbate due to chemical potential difference. In this paper, the decoupling method is adopted, which assumes that the heat exchange process between the bulk gas and the adsorbent is first performed with entropy generation of heat transfer, and then the adsorption phase transition process occurs with entropy generation of adsorption phase change under isothermal conditions.

The entropy generation of isothermal adsorption phase change is caused by the chemical potential difference in the process of non-equilibrium adsorption and can be expressed as [35]:

(19) $$\sigma_{ad}=-\sum_{i=1}^{n}\frac{\left(1-\varepsilon )\rho_{s}\frac{\partial q_{i}}{\partial t}M_{i}(\mu_{g,i}-\mu_{a,i}\right)}{T}$$

where *J_ad,i_* (kg m^−2^ s^−1^) is the thermodynamic flux of *i* component of mass transfer between volume phase and adsorbed phase, *μ_g_* (J kg^−1^) and *μ_a_* (J kg^−1^) is the chemical potential of adsorbate in volume phase and adsorbed phase respectively. The chemical potential of the volume phase can be estimated as:

(20) $$\mu_{g,i}=\mu_{g,i}^{\Theta }+RT_{s}\ln\frac{p_{i}}{p_{i}^{\Theta }}$$

where *μ_g_*^Θ^ (J kg^−1^) is the chemical potential in the standard state. The chemical potential of the adsorbed phase at non-equilibrium state could be estimated approximately as the chemical potential at the saturated (equilibrium) state at the same temperature and amount of adsorbate and thus equal to the chemical potential of the bulk phase gas which in equilibrium with the adsorbed phase [39]. It could be written as [23]:

(21) $$\mu_{a}(p_{i},T_{s},q_{i})\approx \mu_{a}(p_{sa,i},T_{s},q_{i})=\mu_{g}(p_{sa,i},T_{s})=\mu_{g,i}^{\Theta }+RT_{s}\ln\frac{p_{sa,i}}{p_{i}^{\Theta }}$$

where *p_sa,i_* could be back calculated by Toth model:

(22) $$p_{sa,i}=\frac{1}{K_{eq,i}}{\left(\frac{1}{{\left(\frac{q_{i}^{*}}{q_{i}}\right)}^{n_{i}}-1}\right)}^{n_{i}}$$

The entropy generation of heat transfer between gas and solid adsorbent is caused by the temperature difference between the solid adsorbent and the volume phase gas, and can be expressed as:

(23) $$\sigma_{q,sg}=-J_{q,sg}\left(\frac{1}{T}-\frac{1}{T_{s}}\right)=-\frac{(1-\varepsilon )6h_{f}}{d_{p}}\left(T-T_{s}\right)\left(\frac{1}{T}-\frac{1}{T_{s}}\right)$$

The total entropy generation rate is the sum of entropy generation rate of various factors, which can be expressed as:

(24) $$\sigma_{tot}=\sigma_{q,g}+\sigma_{q,s}+\sigma_{diff}+\sigma_{vf}+\sigma_{ad}+\sigma_{q,sg}$$

The total entropy generation of the adsorption separation cycle can be obtained by integrating the entropy generation rate *σ_bed,tot_* of the entire adsorption bed throughout the cycle, which can be expressed by the following equation:

(25) $$\sigma_{g,cycle}=\oint_{cycle}\sigma_{bed,tot}dt=\oint_{cycle}\left({∭}_{bed}\sigma_{tot}dv\right)dt$$

The heat consumption of the TSA cycle is mainly consisting of three part: (1) the sensible heat of the adsorbent caused by increasing temperature. (2) the sensible heat of the adsorbed phase and (3) the latent heat caused by the desorption of the adsorbed gas. And it could be calculated by the following equation [40,41]:

(26) $$E_{t_{1}\sim t_{2}}={\left(E_{SE}+E_{LA}\right)}_{t_{1}\sim t_{2}}$$

(27) $$E_{SE}=(1-\varepsilon )\int_{T_{0}}^{T_{t}}\rho_{s}qC_{p,a}dT+(1-\varepsilon )\int_{T_{0}}^{T_{t}}\rho_{s}C_{p,s}dT$$

(28) $$E_{LA}=(1-\varepsilon )\rho_{s}\sum_{i=1}^{n}\int_{q_{0,i}}^{q_{t,i}}\Delta H_{i}dq_{i}$$

where *E_t_*_1*~t*2_ is the energy consumption from time *t*_1_ to *t*_2_. *T*_0_ and *q*_0_ is the temperature and amount of adsorption at the start of the process, and *T_t_* and *q_t_* is the temperature and amount of adsorption at the end of the process. *C_p,a_* is the specific heat capacity of adsorbed phase which is assumed equal to the specific heat capacity of gas phase.

## 3. Result and Discussion

### 3.1. Mesh Independence

According to conclusions of Ben-Mansour et al. [11], compared with the 1D model, the 2D model and the 3D model have the similar higher accuracy, so this paper adopts the two-dimensional model to save the computing time. The 2D model domain for the adsorbent bed was meshed using structural grid, and the number of grids divided was 720, 1620, 2880 and 4500, respectively. 

**Figure 7:**
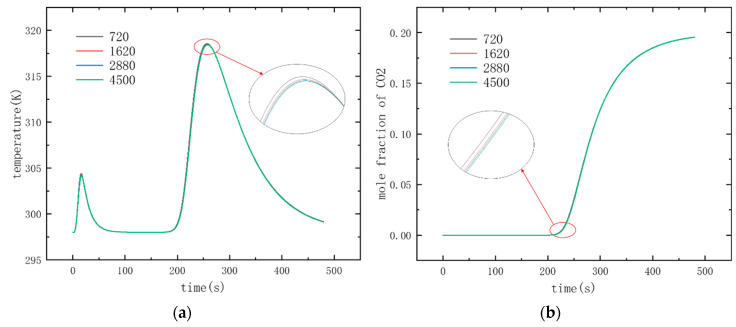
Result of mesh independence analysis: (**a**) temperature profiles at the outlet (**b**) CO_2_/N_2_ concentration ratios at the bed outlet for numerical meshes of 720, 1620, 2880 and 4500 cells.

Four quantities of grids were used for adsorption and breakthrough simulation respectively. The inlet velocity was 0.05 m/s, and the remaining parameters were the values in Table 1; Table 2. The convergence accuracy was set as 10^−6^. The changes of mole fraction and gas temperature at the outlet of adsorption bed with time are calculated as shown in Figure 7, where the simulation results of the four grid numbers are almost the same, which indicates that the grid of any number meets the simulation accuracy. In this paper, mesh number of 2880 are used for the subsequent simulation work.

### 3.2. Entropy Analysis of TSA

**Figure 8:**
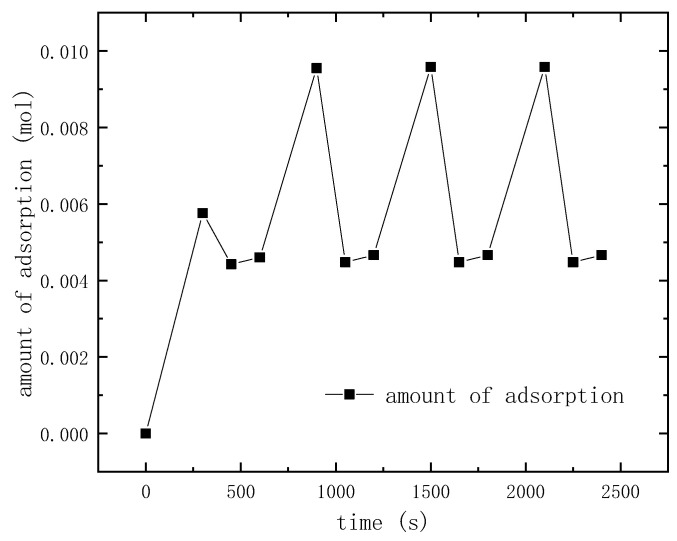
Simulation results of cyclic steady state study.

The changes of adsorption amount over time in five cycles are shown in Figure 8. The results show that the adsorption cycle has reached the steady-state cycle at the fourth cycle, and the relative difference of adsorption amount is less than 1%. In this paper, the irreversibility of the adsorption cycle was analyzed using the data of the fifth cycle.

**Figure 9:**
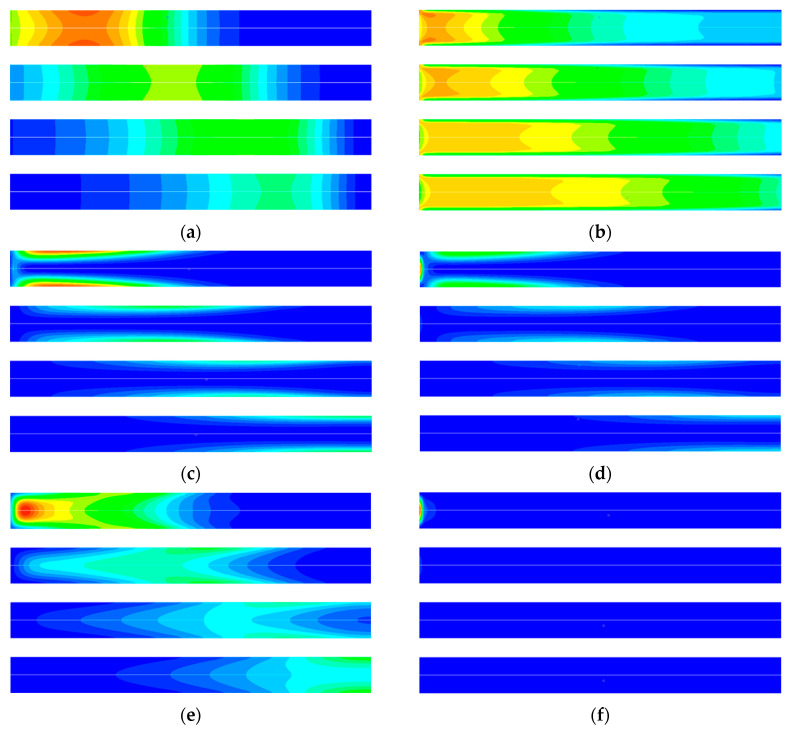
CFD simulation results of entropy generation rate at different time in the adsorption process: (**a**) Entropy generation rate for diffusion 0~20 (J m^−3^ K^−1^). (**b**) Entropy generation rate for viscose flow 0~0.0006 (J m^−3^ K^−1^). (**c**) Entropy generation rate for heat transfer in gas 0~25 (J m^−3^ K^−1^). (**d**) Entropy generation rate for heat transfer in solid 0~8 (J m^−3^ K^−1^). (**e**) Entropy generation rate for adsorption phase change 0~6.5 (J m^−3^ K^−1^). (**f**) Entropy generation rate for heat transfer between gas and solid 0~10.5 (J m^−3^ K^−1^). From top to bottom for each figure, in order, 60 s, 120 s, 180 s, 240 s.

The simulation results of entropy generation in the adsorption process are shown in Figure 9. Figure 9a is the entropy generation rate due to the diffusion of different components of the mixture in the adsorption process. The diffusion entropy generation rate is columnar along the axis. This is because the concentration of CO_2_ decreases along the axis as the CO_2_ in the intake gas is constantly adsorbed, and a large concentration gradient is caused. Figure 9b is the distribution of entropy generation rate generated by viscous friction of the mixture. The entropy generation is connected with the local flow velocity of the mixture in the adsorption bed. Figure 9c,d show the rate of entropy generation in the gas phase and the solid phase due to heat transfer, respectively. It can be seen that heat transfer entropy generation mainly occurs at the inner wall and the inlet of adsorption bed. This is because a large amount of heat will be released in the process of gas adsorption, resulting in a temperature core area at the center of the adsorption bed, and a large temperature gradient will be generated between the axis of the adsorption bed and the inner wall, resulting in a large heat transfer entropy generation rate. Figure 9e shows the entropy generation rate caused by adsorption phase change, which is connected to the adsorption rate and the chemical potential difference between gas phase and adsorption phase. For the adsorbate of unit mass, the greater the chemical potential difference in the adsorption process, the greater the irreversible loss. At the initial stage of the adsorption process, the entropy generation of adsorption was mainly distributed near the inner wall of the adsorption bed, which was due to the large heat loss, low temperature of adsorbent and large adsorption amount caused at the inner wall. With the development of the adsorption process, the temperature at the axis of the adsorption bed decreases, the adsorption rate is greater than that at the inner wall, and the entropy generation rate is larger. Figure 9f is the entropy generation caused by the heat transfer between the gas phase and the solid phase, which is connected to the temperature difference between the adsorbent and the bulk phase gas. The entropy generation generated by heat transfer between the solid phase and the gas phase in the adsorption process is mainly distributed at the inlet of the adsorption bed. This is due to a large amount of heat released during the adsorption process and a temperature core caused by it, resulting in a strong heat transfer between the cold mixture and the gas mixture from the adsorption bed inlet.

**Figure 10:**
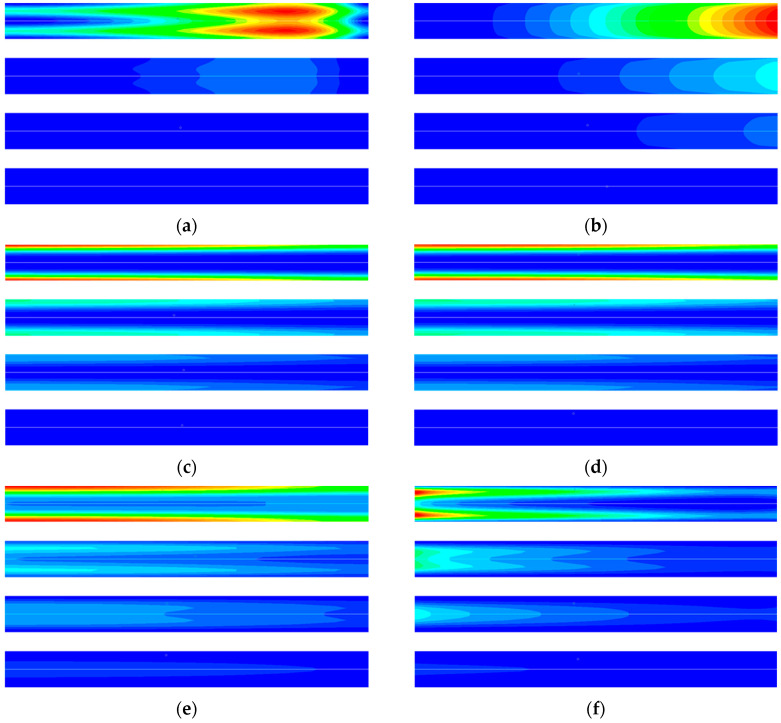
CFD simulation results of entropy generation rate at different time in the desorption process: (**a**) Entropy generation rate for diffusion 0~0.2 (J m^−3^ K^−1^). (**b**) Entropy generation rate for viscose flow 0~0.0021 (J m^−3^ K^−1^). (**c**) Entropy generation rate for heat transfer in gas 0~760 (J m^−3^ K^−1^). (**d**) Entropy generation rate for heat transfer in solid 0~100 (J m^−3^ K^−1^). (**e**) Entropy generation rate for adsorption phase change 0~200 (J m^−3^ K^−1^). (**f**) Entropy generation rate for heat transfer between gas and solid 0~4 (J m^−3^ K^−1^). From top to bottom for each figure, in order, 10 s, 20 s, 30 s, 60 s.

The simulation results of irreversibility in the desorption process are shown in Figure 10. As can be seen from Figure 10a, the entropy generation caused by mixing presents a zonal distribution along the radial direction. This is because the adsorbent near the inner wall of the adsorption bed in the heating process desorbs earlier, and the concentration of CO_2_ decreases along the radial direction, resulting in a concentration gradient that leads to diffusion. The diffusion entropy generation in the desorption process is smaller than that in the adsorption process, because the desorbed gas rapidly diffuses to the axis and cannot form a large concentration gradient radially. Figure 10b shows that entropy generation caused by flow friction is mainly distributed at the outlet of the adsorption bed. The reason is that the desorption gas accumulates continuously along the axial direction, and the outlet has the maximum velocity. Figure 10c,d show the entropy generation caused by heat transfer in the gas phase and solid phase of the desorption process, respectively. It can be seen that the entropy generation is mainly generated in the inner wall of the adsorption bed. This is due to the maximum temperature gradient near the inner wall of the adsorption bed at the beginning of the desorption process. With the development of desorption process, the temperature gradient decreases gradually, and the entropy generation rate caused by it also decreases. Figure 10e is the entropy generation rate caused by adsorption phase change in the desorption process. At the beginning of the desorption process, the entropy generation was mainly distributed near the inner wall of the adsorption bed, which was caused by the large chemical potential difference between the bulk phase gas and the adsorption phase due to the increase of temperature. The chemical potential difference decreases with the desorption process, and the region with the highest entropy generation rate moves gradually toward the axis. According to Figure 10f, the heat transfer entropy generation between gas and solid at the initial stage of the desorption process presents a zonal radial distribution. With the development of desorption process, the entropy generation at the outlet of the adsorption bed decreases gradually. The reason is that the desorbed gas flows along the axis to the outlet and continuously exchanges heat with the adsorbent material, and the temperature difference between the gas-solid phase decreases gradually.

**Figure 11:**
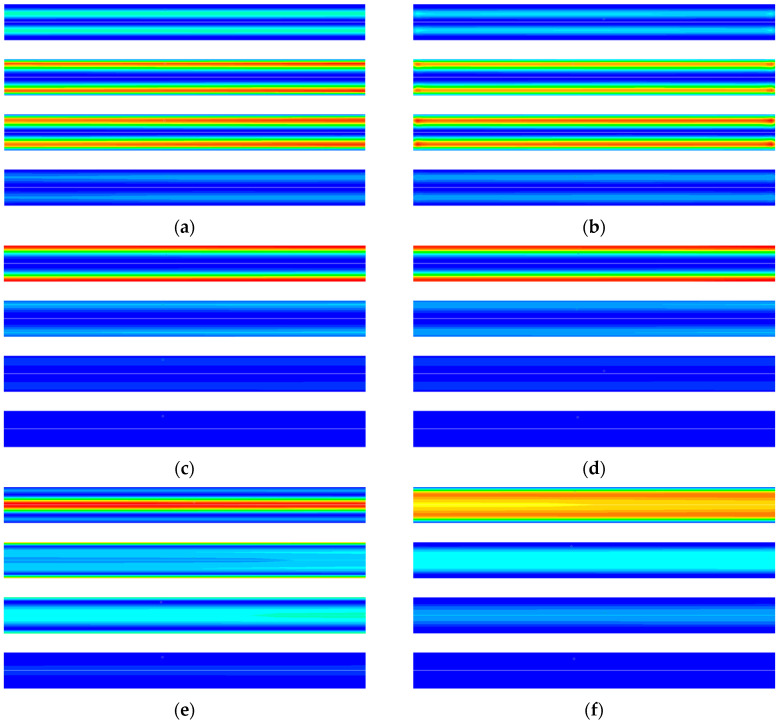
CFD simulation results of entropy generation rate at different time in the cooling process: (**a**) Entropy generation rate for diffusion 0~0.004 (J m^−3^ K^−1^). (**b**) Entropy generation rate for viscose flow 0~5 × 10^−6^ (J m^−3^ K^−1^). (**c**) Entropy generation rate for heat transfer in gas 0~210 (J m^−3^ K^−1^). (**d**) Entropy generation rate for heat transfer in solid 0~28 (J m^−3^ K^−1^). (**e**) Entropy generation rate for adsorption phase change 0~17 (J m^−3^ K^−1^). (**f**) Entropy generation rate for heat transfer between gas and solid 0~1.5 (J m^−3^ K^−1^). From top to bottom for each figure, in order, 10 s, 20 s, 30 s, 60 s.

The cause of irreversibility in the cooling process is similar to that in the desorption process, and the simulation results are shown in Figure 11. From Figure 11a,b, it can be seen that the entropy generation caused by diffusion and flow friction presents a zonal radial distribution. At the beginning of the cooling process, as the adsorbent material near the inner wall of the adsorption bed rapidly cooled and the adsorption capacity increases, the pressure in the adsorption bed also decreases rapidly. The adsorbent material at the center of the adsorption bed begins to desorb, and the desorbed gas flows continuously from the axis to the inner wall of the adsorption bed. Figure 11c,d are the entropy generation due to heat transfer in the gas phase and the solid phase in the desorption process, respectively. It can be seen that the entropy generation is mainly distributed at the inner wall of the adsorption bed. This is due to the large temperature gradient near the inner wall of the adsorption bed during the cooling process. As the desorption process develops, the temperature gradient gradually decreases and the rate of entropy generation decreases. 

**Figure 12:**
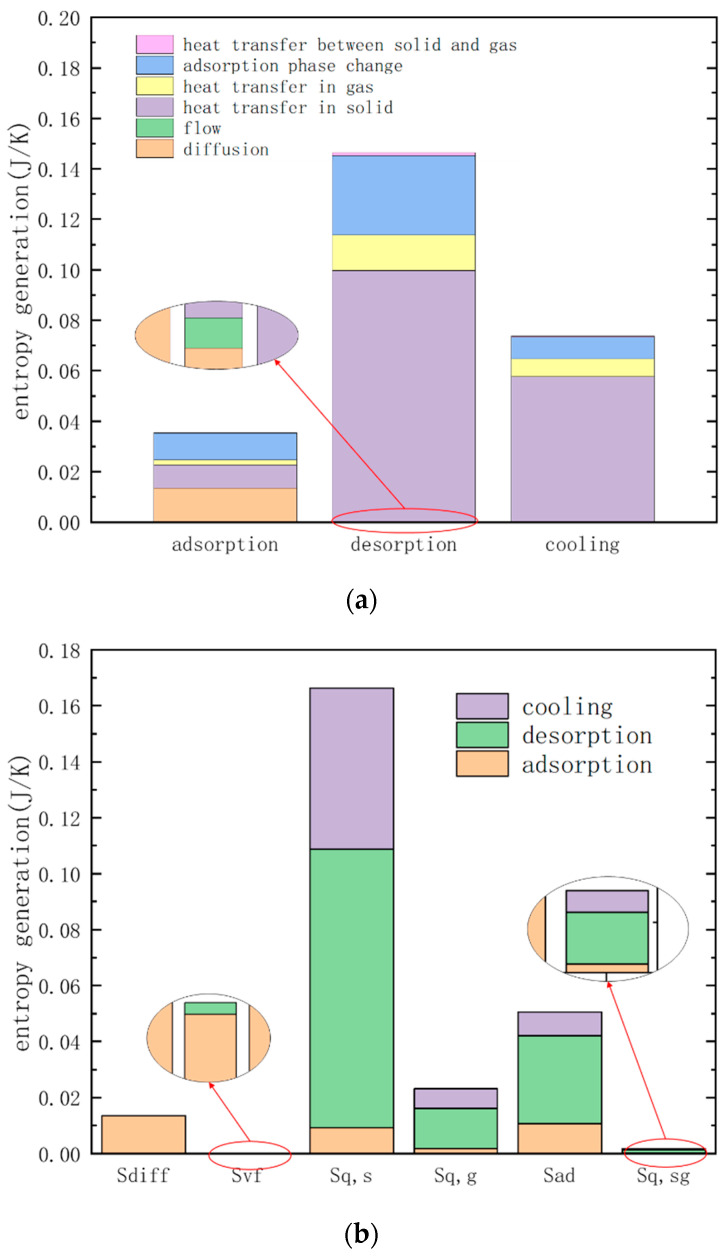
Integral of entropy generation: (**a**) different TSA cycle process (**b**) different Irreversibility factor.

It can be seen from Figure 11e that the entropy generation due to the adsorption phase change is distributed near the inner wall of the adsorption bed and the axis, but the causes of the entropy of the two parts are different. At 10 s, the entropy generation at the inner wall is due to adsorption, and that at the axis is due to the desorption. At 30 s, the temperature at the axis gradually decreases, and the adsorption amount increases, resulting in desorption at the inner wall. Adsorption occurs at the axis, and the adsorbate returns from the inner wall to the axis, the adsorption amount in the adsorption bed gradually becomes uniform. The entropy generation of each process in the TSA cycle can be obtained by the integral method, and the calculation result is shown in Figure 12a. The integral result of the entropy generation of various factors is shown in Figure 12b. It can be seen from the figure that the entropy produced by irreversible heat transfer accounts for the largest proportion, approaches 70%, and the entropy generation by adsorption phase change and diffusion ranks second and third respectively.

In addition, due to the slower flow rate in the adsorption bed during the adsorption process, the irreversible loss caused by viscous friction is so small that it can be ignored. The gas-solid heat transfer entropy is also very small, that is because the heat exchange is intense due to sufficient contact between the gas phase and the solid phase, and the temperature difference between the gas phase and the solid phase and the entropy produced by it are also negligible. The entropy generation caused by heat transfer during desorption and cooling accounts for a large proportion, while the degree of irreversibility caused by diffusion, heat transfer and adsorption phase change in the adsorption process is equivalent. Therefore, the reduction of the irreversible loss of the adsorption separation cycle should be mainly focus on the irreversible loss of heat transfer, adsorption and diffusion.

### 3.3. Parameter Analysis of Heating Process

In this part, the heating desorption process is taken as an example for parameter analysis, and the cause of irreversibility in the adsorption bed and possible optimization directions are further studied. Since the entropy of diffusion and the viscos friction of the desorption process account for a small proportion, and the entropy generation of diffusion is difficult to reduce by changing the parameters in the adsorption process, this section focuses on the effects of changing the thermal properties of the heat-transfer-related and the adsorption-related parameters on the irreversibility of the desorption process.

#### 3.3.1. Thermal Conductivity of the Adsorbent

**Figure 13:**
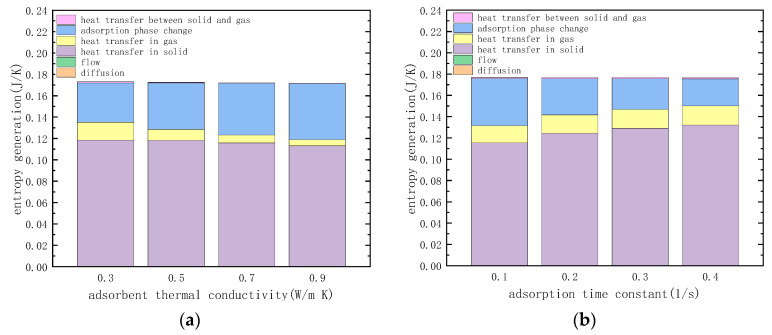
Entropy generation with different adsorbent thermal conductivity of adsorbent and adsorption time constant of adsorbent: (**a**) different adsorbent thermal conductivity of adsorbent (**b**) different adsorption time constant of adsorbent.

The entropy generation of various irreversible factors in the heating desorption process was calculated with the thermal conductivity coefficients of 0.3, 0.5, 0.7 and 0.9 (W m^−1^ K^−1^), respectively, and the simulation results are shown in Figure 13a. It shows that increasing the thermal conductivity of the adsorbent material will reduce the entropy generation of heat transfer, but the entropy generation of adsorption phase change increases, and the total entropy generation is almost unchanged. This is because the increase in the thermal conductivity of the adsorbent material causes the temperature gradient within the sorbent material packed bed to decrease, resulting in a decrease in the irreversibility of the transfer of the same amount of heat. However, increasing the thermal conductivity increases the ability of the adsorbent layer to transfer temperature, and the chemical potential difference that drives the adsorption phase change increases, resulting in an increase in the irreversibility of the adsorption phase change.

#### 3.3.2. Adsorption Time Constant

The entropy generation of various irreversible factors in the heating desorption process was calculated under the condition that the adsorption time constants of CO_2_ and N_2_ were 0.1, 0.2, 0.3 and 0.4 (s^−1^), respectively, and the calculation results are shown in Figure 13b. It shows that increasing the adsorption time constant of the adsorbent material leads to a decrease in the entropy generation of adsorption phase change, but it leads to an increase in entropy generation of heat transfer, and the total entropy generation is almost unchanged. This is because the increase in the adsorption time constant of the adsorbent material leads to a decrease in the chemical potential difference that promotes the adsorption phase change, and the irreversibility of the adsorption of the same mass of adsorbate decreases. However, increasing the adsorption time constant will increase the adsorption phase transition phenomenon, which will result in a decrease in the temperature transfer capability in the adsorbent packed bed, which in turn leads to a larger temperature gradient and an increase in irreversibility.

The comparison of the thermal conductivity and adsorption time constant of different adsorbent materials shows that the thermal conductivity and adsorption time constant of the adsorbent material alone have an effect on both irreversible losses. This indicates that the heat transfer and adsorption phase change in the adsorbent bed are coupled with each other. In practice, the design of the physical properties of the adsorbent material should fully consider the influence on different irreversibility.

#### 3.3.3. Specific Heat Capacity of the Adsorbent

**Figure 14:**
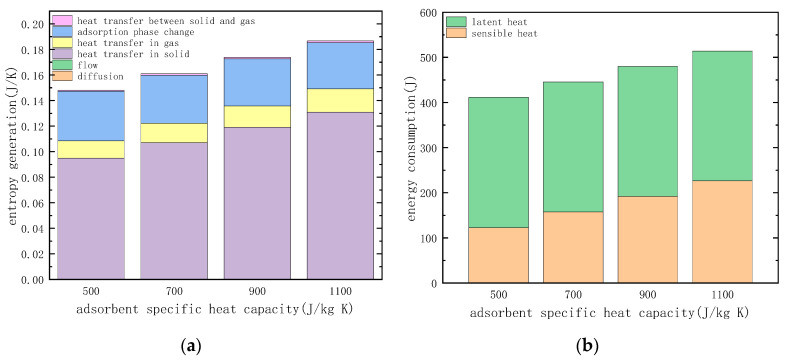
Effect of adsorbent specific heat capacity: (**a**) entropy generation of different factors. (**b**) energy consumption in the desorption process.

The entropy generation of various irreversible factors in the heating desorption process was calculated with specific heat capacities of 500, 700, 900 and 1100 (J kg^−1^ K^−1^), respectively, and the calculation results are shown in Figure 14a. The change in the specific heat capacity of the adsorbent also causes a change in the amount of heat in the process of desorption. The calculation of the heat required for the desorption process is shown in Figure 14b.

As can be seen from Figure 14a, reducing the specific heat capacity of the adsorbent material will reduces the entropy generation of heat transfer, and it can be seen from Figure 14b that the heat consumption in the desorption process is also reduced. This is due to the fact that the heat transferred in the radial direction of the desorption process is reduced, the temperature transfer capability is enhanced, and the temperature gradient and the entropy generation caused are reduced. In addition, reducing the specific heat capacity of the adsorbent material can reduce the sensible heat needed for the adsorbent. Therefore, the adsorbent material should be designed to minimize the specific heat capacity with the amount of adsorption unchanged, thus reducing the heat consumption and the irreversible loss during desorption.

#### 3.3.4. Temperature of Desorption

**Figure 15:**
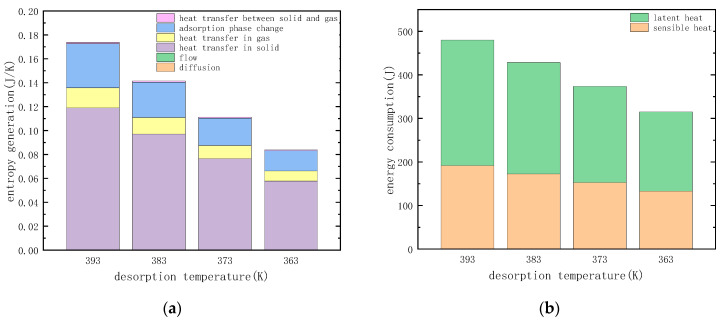
Effect of temperature of desorption: (**a**) entropy generation of different factors. (**b**) energy consumption in the desorption process.

The entropy generation of various irreversible factors in the heating desorption process was calculated under the conditions of desorption temperatures of 393, 383, 373 and 363 (K), respectively, and the calculation results are shown in Figure 15a. The change in desorption temperature also causes the heat consumption in the process of desorption to change, and the heat required for the desorption process is shown in Figure 15b.

As can be seen from Figure 15a, the reduction of the desorption temperature causes the entropy generation of heat transfer to decrease, and it can be seen from Figure 15b that the desorption heat consumption is also reduced. As the desorption temperature is reduced from 393 K to 363 K, the heat consumption during the desorption process is reduced by nearly 30%. The entropy generation is reduced by nearly 50%, which is greater than the reduction in heat consumption during the desorption process. This is because the decrease in the desorption temperature causes the temperature difference that drives the heat transfer process to decrease. It is noted that reducing the desorption temperature also reduces the irreversibility of the adsorption phase change. This is because the temperature gradient of the adsorbent material is reduced due to the decrease in temperature difference, and the chemical potential difference in the adsorption phase transition process is also reduced.

For the TSA cycle, the temperature difference between the high and low temperature heat sources is the fundamental driving force for the TSA cycle. In the initial stage of the desorption process and the cooling process, a large irreversible loss occurs due to the large temperature difference between the heat source and the adsorption bed. As the process develops, the thermodynamic forces that drive the process will continue to decrease and the irreversible losses will also decrease. Similar conclusions can be drawn from the literature [26,27,28]. Therefore, reducing the irreversible loss of the TSA cycle can be started from matching the different grade heat sources with the adsorption bed of different temperature during the TSA cycle. This conclusion can be further derived from the entropy analysis of the regenerative TSA cycle in the next section.

### 3.4. TSA Cycle with Heat Regeneration

**Figure 16:**
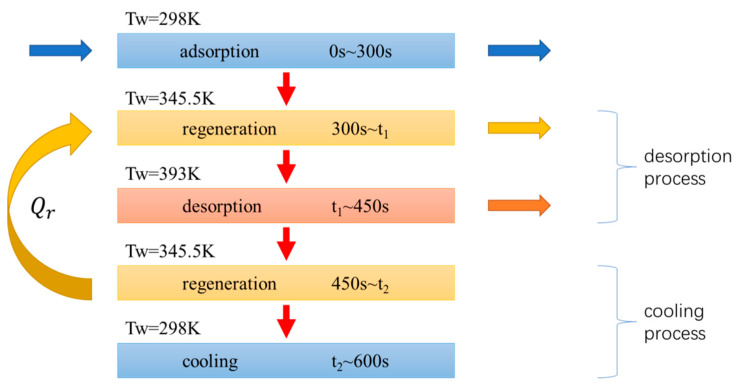
Schematic diagram regenerative cycle of single adsorption bed.

The desorption process of the regenerative cycle used in this paper consists of two parts, including the regenerative process and the heating process accomplished by external heat source. The cooling process consists of a regenerative process and a cooling process accomplished by external heat sink. For a dual bed cycle, the heat recovered during the cooling process is used for the heating of the adsorbent bed desorption process by a regeneration, which can be accomplished by continuously flowing intermediate temperature fluid through the desorption bed and the cooling bed. For a single column regenerative cycle, unlike that of a double column, the cycle process can be expressed as storing the heat released by the cooling process in the intermediate temperature fluid for heating the cycle process, as shown in Figure 16.

It is assumed that the intermediate temperature fluid used for regeneration has a very fast flow rate and the heat capacity is very large, resulting in the temperature of the fluid is approximately constant during the regenerative process. The intermediate temperature *T_m_* is calculated using the average of the adsorption temperature and the desorption temperature, which can be expressed by Equation (29):

(29) $$T_{m}=(T_{ads}+T_{des})/2$$

The fraction of heat recovered is defined by the ratio of the recovered heat *Q_r_* and the released heat in the process of desorption without regeneration, and can be expressed by Equation (30):

(30) $$\alpha =\frac{Q_{r}}{Q_{cool}}=\frac{E_{300\sim t_{1},r}}{E_{450\sim 600}}=\frac{E_{450\sim t2,r}}{E_{450\sim 600}}$$

where *E*_300*~t*1*,r*_ (J) is the heat consumption in the process of regeneration of the desorption process, *E*_450*~t*2*,r*_ (J) is the heat released from the adsorption bed during the regeneration process in the process of cooling and is equal to *E*_300*~t*1*,r*_. *E*_450*~*600_ (J) is the heat released by the adsorption bed during the cooling process in the cycle without heat regeneration. The times of heat regeneration *t*_1_ and *t*_2_ can be determined by the fraction of heat recovered *α*.

The specific entropy generation *s_g,TSA_* and specific energy consumption *e_TSA_* of the cycle are the entropy generation and energy consumption of CO_2_ captured for unit mass, which can be expressed by Equations (22) and (23):

(31) $$s_{g,TSA}=S_{TSA}/m_{CO_{2}}$$

(32) $$e_{TSA}=E_{TSA}/m_{CO_{2}}$$

The purity of the CO_2_ captured is the ratio of the mass of CO_2_ to the mass of the product gas, which can be expressed by Equation (33):

(33) $$Puirty=m_{CO_{2}}/(m_{CO_{2}}+m_{N_{2}})$$

where *S_TSA_* (J K^−1^) and *E_TSA_* (J) is the entropy generation and heat consumption of cycle, respectively. And *m_CO_*_2_ is the CO_2_ captured of the cycle. When the fraction of heat recovered is set to 45%, the specific energy consumption and specific entropy generation with or without heat recovery are calculated as shown in Table 4.

**Figure 17:**
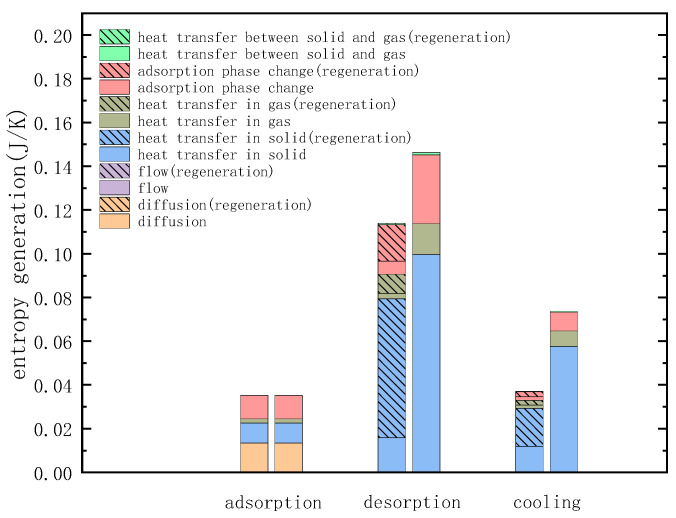
Comparison of entropy generation with or without regeneration process. (left column of each process): entropy generation with regeneration; (right column of each process): entropy generation without regeneration.

The result of entropy generation calculated with the presence or absence of the regenerative cycle is shown in Figure 17.

From the calculation results of the cycle performance in Table 4, it is known that the specific heat consumption of the cycle with regeneration is 12.2% lower than that of the cycle without that process, and the entropy generation is reduced by 19.4%. The reductions in the irreversible loss of desorption and cooling process is much greater than the reduction in specific energy consumption, which is close to twice of it. It can be seen from Figure 17 that the entropy generation of heat transfer and the adsorption phase change in the regenerative cycle are reduced compared to the cycle that without regeneration. This is because when the cycle has a regeneration process, the presence of the intermediate temperature causes the temperature difference of the cycle to decrease (the temperature difference is reduced to half of the cycle without regeneration), and the chemical potential difference of the adsorption phase change also decreases, this reduces the irreversible loss of heat transfer and adsorption phase change. The amount of CO_2_ captured in the cycle with regeneration is slightly reduced compared to the case without regeneration. This is because the temperature difference between the heat transfer fluid and the adsorbed bed is reduced and the degree of desorption is slightly decreased with the regeneration process is divided into two processes with the total desorption time unchanged.

It can be seen from the above calculation results that for the TSA cycle, the use of regenerative process can not only make full use of the heat released during the cooling process, reduce the energy consumption during the desorption process, but also, can reduce the irreversible loss of the cycle, improve the energy utilization efficiency. The most important reason is because it allows a more efficient use of heat of different grades in the cycle, and the temperature between the heat source and the adsorbent bed can be better matched. Therefore, for the adsorption separation cycle in engineering practice, a regenerative cycle should be used as much as possible to improve the efficiency of energy utilization.

## 4. Conclusions

In this paper, the CFD method is used to calculate and analyze the entropy generation of different TSA cycle processes. The mechanism of entropy generation and the measures to reduce entropy generation under non-equilibrium conditions are discussed. The entropy generation of heat transfer in the TSA cycle accounts for a largest proportion, followed by the entropy generation of adsorption phase change and diffusion. The entropy generation of heat transfer between gas and solid and viscous flow is small and can be ignored. The entropy generation rate distribution and changes with time is also analyzed.

The desorption process is taken as an example to conduct the sensitivity analyze of the main irreversible factors of the TSA process, including the specific heat capacity of the adsorbent, the adsorption time constant, the thermal conductivity of the adsorbent and the desorption temperature. Increasing the thermal conductivity of the adsorbent and reducing the specific heat capacity of the adsorbent can enhance the heat transfer and reduce the irreversible loss. Increasing the adsorption time constant of the adsorbent can reduce the chemical potential difference and corresponding irreversible loss. The heat transfer and adsorption phase change process coupled with each other and the design of the physical properties of the adsorbent material should fully consider the influence on different irreversibility.

The temperature difference between the adsorption temperature and the desorption temperature is the direct driving force of the TSA cycle, but the existence of the temperature difference causes irreversible heat transfer loss. In engineering practice, the heat transfer temperature difference and the irreversible loss caused by it should be reduced as much as possible with the amount of CO_2_ captured little changed, and matching the different grade heat sources with the adsorption bed of different temperature during the TSA cycle. The use of the regenerative process can not only reduce the energy consumption of the TSA cycle, but also reduce the irreversible loss by reducing the temperature difference and improve the energy utilization efficiency. In summary, the entropy analysis of the TSA cycle can provide guidance for the engineering practice and improvement of energy-efficiency.

## Figures and Tables

**Table 1:** Properties of the adsorbed bed used in CFD modeling [11].

Parameters	Value	Unit
Length of adsorption bed, *L*	10	cm
Inner diameter of adsorption bed, *D*	1	cm
Porosity, *ε*	0.7417	
Adsorbent specific heat capacity, *C_p,s_*	911	J/kg K
Absorbent thermal conductivity, *k_s_*	900	W/m K
Inlet velocity, *v_in_*	0.03	m/s
Inlet mole fraction, *x_in_*	0.2	
Adsorption temperature, *T_ads_*	298	K
Desorption temperature, *T_des_*	393	K
Operation pressure, *p_b_*	101,300	Pa

**Table 2:** Properties of the adsorbent used in CFD modeling [11].

	*q_m_* (mol kg^−1^)	*K*_0_ (Pa^−1^)	*n*	Δ*H* (kJ mol^−1^)	*K_L_* (s^−1^)
CO_2_	11.4048	3.089 × 10^−11^	0.4217	−42,000	0.1182
N_2_	6.7072	9.36 × 10^−10^	1	−18,000	0.3043

**Table 3:** Summary of irreversible losses in the adsorption process.

Different Factors of Entropy Generation	Cause of Entropy Generation
Entropy generation of heat transfer	1. Entropy generation of heat transfer in solids 2. Entropy generation of heat transfer in gas 3. Entropy generation of heat transfer between gas and solid
Entropy generation of diffusion	1. Entropy generation of diffusion between different components of the mixture
Entropy generation of flow friction	1. Entropy generation of flow friction dissipation between the wall and the mixture 2. Entropy generation of flow friction dissipation between adsorbent particles and mixture
Entropy generation of phase change	1. Entropy generation of adsorption phase change between adsorbent and mixture

**Table 4:** Comparison of the performance of regenerative cycles.

Cycle	Productivity of CO_2_ (kg)	Specific Energy Consumption (GJ t^−1^)	Specific Entropy Generation (J kg^−1^)	Purity of Product Gas
Cycle with regeneration	0.000202	1.672	924	0.842
Cycle without regeneration	0.000223	1.906	1147	0.844

## Nomenclature

Symbol	
*C*_2_	inertial resistance factor, m^−1^
*C_p,s_*	adsorbent specific heat capacity, J kg^−1^ k^−1^
*d_p_*	particle diameter, m
*D*	diameter, m
*D_i,m_*	effective diffusion coefficient for component *i*, m^2^ s^−1^
*D_i,j_*	diffusion coefficient of component *i* in component *j*, m^2^ s^−1^
*E*	total energy of gas, J m^−3^
*E*_*t*1~*t*2_	energy consumption between *t*_1_ and *t*_2_
*h*	enthalpy
*h_f_*	heat transfer coefficient between gas phase and solid, W m^−2^ K^−1^
∆*H*	the heat of adsorption, J mol^−1^
*J*	diffusion flux, kg m^−2^ s^−1^
*k_eq_*	isotherm adsorption constant, Pa^−1^
*k*_0_	adsorption constant at infinite dilution, Pa^−1^
*k_L_*	LDF adsorption time constant, s^−1^
*k*	thermal conductivity of gas
*k_s_*	adsorbent thermal conductivity, W m^−1^ K^−1^
*L*	length, m
*M*	molecular weight, mol kg^−1^
*m*	mass of captured, kg
*n*	heterogeneity parameter
*p*	Pressure, Pa
*q*	adsorption uptake, mol kg^−1^
*q**	equilibrium adsorption uptake, mol kg^−1^
*q_m_*	maximum adsorption uptake, mol kg^−1^
*R*	universal gas constant, J mol^−1^ K^−1^
*Sg*	entropy generation, J K^−1^
*S_momentum_*	momentum source term
*T*	temperature, K
*t*	time, s
*x*	mole fraction
*y*	mass fraction
Greek letters	
*α*	permeability m^2^
*ε*	porosity
*ρ*	density, kg m^−3^
*σ*	entropy generation rate, W m^3^ K^−1^
*μ*	gas dynamic viscosity, Pa s
*μ_a_*	chemical potential of adsorbed phase, J kg^−1^
*μ_g_*	chemical potential of gas, J kg^−1^
*δ*	bed wall thickness, m
*τ*	stress tensor
*χ*	chemical potential of gas in diffusion process, J kg^−1^
Subscripts	
a	adsorbed phase
ad	adsorption
b	bed
c	critical
d	diffusion
g	gas
i	components
in	inlet
p	particle
q	heat transfer

## Appendix A

This part introduces the calculation method of gas physical properties, and the details are shown below.

The specific heat capacity of the gas mixture is determined by the following mixing-law:

(A1) $$C_{p}=\sum_{i=1}^{n}y_{i}C_{p,i}$$

The thermal conductivity of the gas mixture is determined by the following mass-weight-mixing-law:

(A2) $$k=\sum_{i}\frac{x_{i}k_{i}}{\sum_{j}x_{j}\phi_{i,j}}$$

Where:

(A3) $$\phi_{i,j}=\frac{{\left[1+{\left(\frac{\mu_{i}}{\mu_{j}}\right)}^{1/2}{\left(\frac{M_{W,i}}{M_{W,j}}\right)}^{1/4}\right]}^{2}}{{\left[8\left(1+\frac{M_{W,i}}{M_{W,j}}\right)\right]}^{1/2}}$$

The viscosity of the gas mixture is determined by the following mass-weight-mixing-law:

(A4) $$\mu =\sum_{i}\frac{x_{i}\mu_{i}}{\sum_{j}x_{j}\phi_{i,j}}$$
